# The Clue is in the Eyes. A Case Report of Internuclear Ophthalmoplegia

**DOI:** 10.21980/J8DP9M

**Published:** 2024-01-31

**Authors:** Cooper Nickels, Christy Keyes, Caroline Astemborski, Haley Fulton

**Affiliations:** * Prisma Health Upstate, Department of Emergency Medicine, Greenville, South Carolina; ^ University of South Carolina School of Medicine Greenville (USCSOMG), Greenville, South Carolina

## Abstract

The chief complaint of vertiginous symptoms can be daunting, and the differential is quite long. Approximately 15% of patients presenting to the emergency department (ED) with dizziness have a dangerous underlying cause.^1^ We present a case of a 40-year-old female with a sudden onset of what she describes as vertigo, in the setting of intermittent diplopia. The patient was found to have a left medial rectus palsy consistent with a left internuclear ophthalmoplegia. Internuclear ophthalmoplegia (INO) is an abnormal gaze that is characterized by the weakness or inability to adduct the affected eye. This occurs secondary to a lesion in the brain affecting the medial longitudinal fasciculus (MLF) most commonly in the pons; however, this pathway can also be affected in the midbrain.^2^ The diagnosis in our patient was confirmed after an MRI revealed an acute infarct of the left dorsal pons involving the medial longitudinal fasciculus, resulting in the observed left INO. The patient was admitted to the hospitalist service with neurology consultation for further stroke workup. Ultimately, the stroke was deemed cryptogenic in etiology with hyperlipidemia and obesity as the patient’s risk factors. She was discharged home after three days in the hospital on daily aspirin and high-intensity statin. Upon six-month follow up she had near resolution of her symptoms.

**Topics:**

Internuclear Ophthalmoplegia, INO, Vertigo, Stroke, Neurology

[Fig f2-jetem-9-1-v1] [Fig f3-jetem-9-1-v1] [Fig f4-jetem-9-1-v1]

**Figure f2-jetem-9-1-v1:**
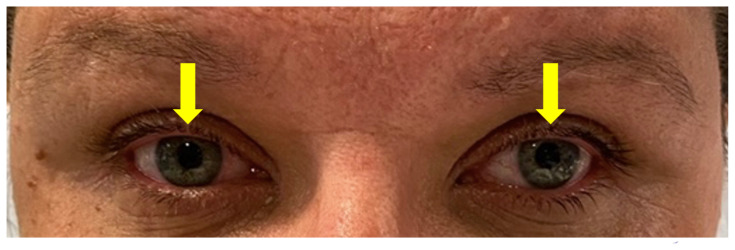


**Figure f3-jetem-9-1-v1:**
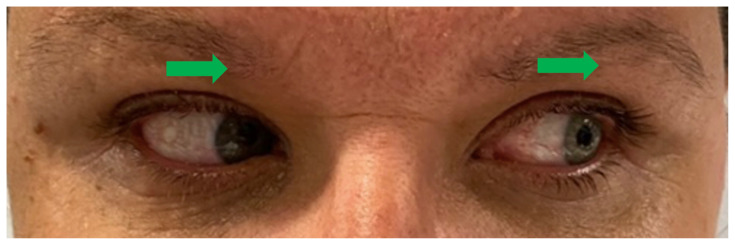


**Figure f4-jetem-9-1-v1:**
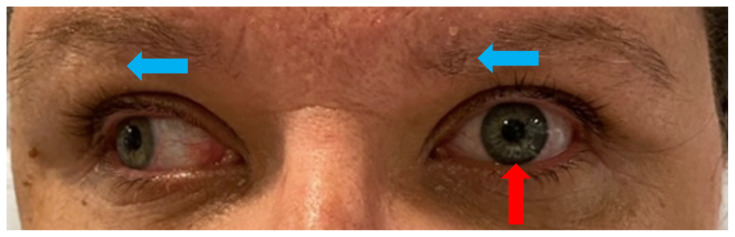


## Brief introduction

Internuclear ophthalmoplegia (INO) is an abnormal gaze that is characterized by the weakness or inability to adduct the affected eye. The abnormal gaze occurs secondary to a lesion within the brain, affecting the medial longitudinal fasciculus (MLF) most commonly in the pons; however, this pathway can also be affected in the midbrain.^2^ The most common etiologies are secondary to ischemia from cerebrovascular accident, or demyelination from multiple sclerosis. Together these account for more than 70% of cases of INO.^3^,^4^ INO secondary to infarction classically presents in older patients with an average age of 62–66.^4^,^5^ The resultant symptoms from the patient’s inability to adduct their eye are diplopia, loss of depth perception, and the report of vertigo.^4^ Workup should include an MRI brain with and without contrast. Prognosis is variable depending on the underlying etiology, but deficits will typically resolve within months; however, they can be long-standing.^5^,^6^

Here, we will discuss a case of a rather young individual, a 40-year-old, in the age range for demyelination etiology and much younger than the average age for ischemic etiology, with a unilateral internuclear ophthalmoplegia who presented with vertigo as her main complaint. She was ultimately found to have an acute infarct on MRI within the left pons, specifically the medial longitudinal fasciculus, resulting in the observed left internuclear ophthalmoplegia. The case is accompanied with clinical pictures to aid with learning and teaching. We will then further discuss the pathophysiology, etiology, prognosis, and treatment of INO. Patient written informed consent for medical photography was obtained during the patient’s time in the emergency department. Approval was granted by the hospital’s institutional board review (IRB) committee for publication and distribution of this case.

## Presenting concerns and clinical findings

A 40-year-old female presented to the ED with a chief complaint of “dizziness.” She reported no significant past medical history. However, approximately three years prior to this ED visit, she had vertiginous symptoms and was evaluated in the ED in conjunction with neurology. At that time, she was noted to have bilateral nystagmus. Computed tomography angiogram head and neck and MRI brain with and without contrast were all obtained at that time, which were all unremarkable. After several rounds of antiemetics and an observation period, patient’s symptoms resolved. She was diagnosed with labyrinthine vertigo, and discharged home with meclizine and primary care follow-up. Since that time, she has had occasional intermittent vertigo typically lasting seconds to minutes in duration, worse with certain movements or positions.

On the day of the presentation, while eating breakfast and watching television, the patient developed sudden onset of what she described as vertigo; unsteadiness on her feet to the point where she had to hold on to objects near her for assistance with associated nausea. She believed her symptoms were reminiscent of her severe vertigo event approximately three years ago. She attempted relief with meclizine without any improvement. Her symptoms resolved by not moving her head or eyes. A review of systems was positive for intermittent double vision, but negative for blurry vision, dysarthria, dysphagia, weakness, numbness, tingling. She denied a history of intermittent symptoms of brief neurologic dysfunction, such as numbness, weakness, visual changes, electrical shocking sensation, urinary incontinence, personal history of autoimmune diseases, family history of autoimmune diseases including multiple sclerosis. Her social history was negative for tobacco use. Her family history was significant for maternal grandmother who suffered a large ischemic stroke at the age of 32 of cryptogenic etiology.

## Significant findings

Upon arrival to the ED the patient was afebrile at 97.8 °F (36.6 °C), heart rate 73 bpm, blood pressure 145/81 mm/Hg, respiratory rate 18/min, and weight 98 kg. On examination, she was in no acute distress, holding an emesis bag in her hand. Her speech was clear and fluent without aphasia or dysarthria. Heart with regular rate and rhythm without murmurs. Lungs clear to auscultation. Abdomen soft and non-tender. Muscle strength was 5/5 diffusely in all four extremities. Sensation intact in all four extremities. No truncal ataxia. Normal heel-to-shin and finger-to-nose bilaterally. Pupils equal round and reactive to light. No ptosis. There was no appreciable esotropia or exotropia noted on straight gaze (yellow arrows). On extraocular muscle examination, patient was noted to have a complete left medial rectus palsy consistent with a left internuclear ophthalmoplegia (red arrow). This was evidence by both eyes easily gazing left (green arrows); however, with rightward gaze, her left eye failed to gaze past midline (red arrow). This rightward gaze recreated her diplopia and reported symptoms of vertigo. There was no nystagmus at rest; however, there was right eye horizontal nystagmus with abduction. Patient’s binocular diplopia resolved by individually covering each eye. Visual fields were full. All other cranial nerves were intact. Physical exam otherwise unremarkable.

## Patient course

Major consideration was given to demyelinating disease versus brainstem infarction as the cause of the INO in this patient given her age. However, the INO was unilateral which favored brainstem infarction. Stroke alert was not activated given low NIH Stroke Scale. We also considered alternative etiologies such as local compression from hematoma; however, there were no reports of any trauma. Nor were there risk factors or a history consistent with a brainstem tumor. The case was discussed with neurology, who recommended an MRI and agreed with withholding on stroke alert. EKG demonstrated a normal sinus rhythm with nonspecific interventricular conduction delay. Complete blood count and complete metabolic panel were unremarkable. Brain MRI with and without contrast revealed an acute infarction of the left dorsal pons involving the medial longitudinal fasciculus. Imaging was consistent with the observed left internuclear ophthalmoplegia. Patient was given 324 mg aspirin and was admitted to the hospitalist service with neurology consultation for further stroke work up and management.

While inpatient, further studies were performed including CT angiogram head and neck which did not reveal any stenosis or arterial abnormality. Transthoracic echocardiogram with bubble study demonstrated a normal ejection fraction at 55–65% with no evidence of a patent foramen ovale. Lipid panel revealed elevated LDL at 141 mg/dL, but otherwise reassuring. Hemoglobin A1c was reassuring at 4.7%. Hypercoagulable panel was negative. Blood pressure remained less than 130 mmHg systolic without intervention during hospital course. Patient was discharged home on hospital day three on long term high intensity statin, Lipitor 80 mg daily, and aspirin 81 mg daily, educated on diet and lifestyle management with LDL goal <70 mg/dL, given a 30-day cardiac event monitor. She was referred for follow up with neurology, ophthalmology, cardiology, and PM&R young stroke rehabilitation as an outpatient.

At six-month follow up, patient had near resolution of her symptoms, with occasional left sided double vision with rapid head movement. She continued her daily aspirin and Lipitor. She followed up with ophthalmology following her hospital stay and was given a prism lens to assist her vision, but only had to wear these for a few days as her visual symptoms started to improve. Her 30-day event monitor was unremarkable. She underwent a transesophageal echocardiogram (TEE) with cardiology which was negative for an intracardiac shunt. There were no adverse or unanticipated events. Ultimately, the cause of her stroke was cryptogenic, with her dyslipidemia and obesity being her risk factors.

## Discussion

**Figure 1:**
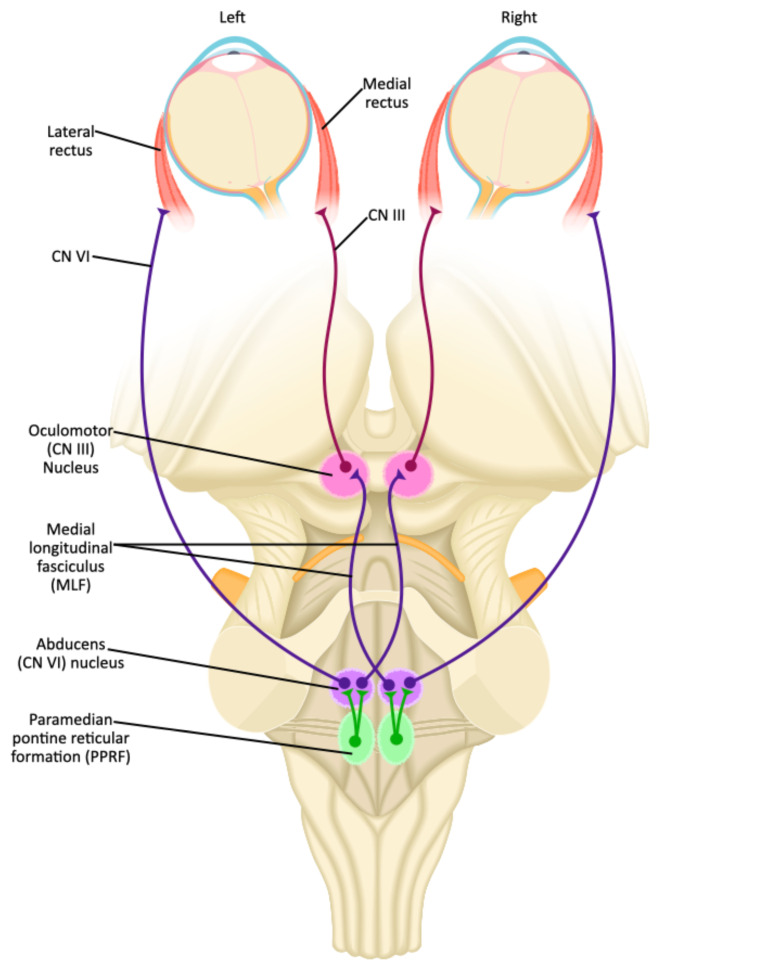
JETem Created Image adapted from Togopic. Brainstem (dorsal view). In: Wikimedia Commons. https://commons.wikimedia.org/wiki/File:202104_Brainstem_dorsal_view.svg Published April 1, 2021. Accessed October 12, 2023. CC BY-SA 4.0.

The neuroanatomy for ocular motion is complex, especially when discussing the conjugate horizontal gaze and the medial longitudinal fasciculus (MLF) pathway. First, the initiation of horizontal eye movement occurs within the frontal eye fields contralateral to the direction of the desired gaze.^3^,^7^ This signal is transmitted to the contralateral cranial nerve (CN) VI nucleus via the paramedian pontine reticular formation (PPRF) (Figure 1).^7^ The CN VI nucleus directly innervates the ipsilateral lateral rectus muscle. At the same time, nerve fibers from CN VI nucleus also send innervation to the contralateral CN III nucleus, which is located in the midbrain, by way of the medial longitudinal fasciculus, which subsequently innervates the respective medial rectus muscle (Figure 1).^7^ Together, contraction of the ipsilateral lateral rectus and contralateral medial rectus results in conjugate horizontal gaze in the desired direction. In addition to coordinating synchronous horizontal eye movements, the MLF is also involved aligning the eyes and assists with vertical pursuits.^7^

Internuclear ophthalmoplegia (INO), also known as internuclear ophthalmoparesis, is an abnormal gaze that is characterized by the weakness or the inability to adduct the affected eye. It occurs secondary to a lesion in the brain that affects the medial longitudinal fasciculus (MLF). Lesions to the MLF occur more commonly in the pons, but also occur in the midbrain.^2^ An INO can be unilateral or bilateral. Unilateral INOs are named for the side that the adduction deficit is on. The dysconjugate movement of horizontal gaze, from the weakness or lack of function of the medial rectus muscle, results in patients experiencing diplopia, loss of depth perception, and visual confusion.^9^ Secondary to these symptoms, it is not uncommon to have patients report vertigo.^4^ Patients will also notice their symptoms more when turning their head or looking in one direction, which accentuates the deficient. These symptoms put patients at risk for falls and motor vehicle accidents.^4^,^9^

On examination, patients will have weakness or complete loss of adduction depending on the cause and severity of the INO. The abducting eye will typically demonstrate horizontal nystagmus.^10^ In addition, some patients might demonstrate abduction slowing of the affected eye.^10^,^11^ This is thought to be due to defective relaxation of the antagonist.^12^ Convergence will be preserved in an isolated INO. This helps to distinguish an INO from a partial CN III palsy. In addition to the INO varying from being subtle to obvious, as well as unilateral or bilateral, it is also important to remember that the patient’s only deficit may be the INO, or it could be one of multiple deficits depending on the cause and extent of the disease.

The differential diagnosis for internuclear ophthalmoplegia is broad (table 1), but the two most common causes, which together account for greater than 70% of cases, are multiple sclerosis and cerebrovascular disease.^3^ In fact according to Frohman et al., INO is the most common eye movement abnormality in patients with multiple sclerosis.^9^ Internuclear ophthalmoplegia caused by multiple sclerosis classically presents in a younger patient population, usually in patients under 45 years old. Furthermore, in patients with multiple sclerosis it is more likely to present bilaterally.^9^ This contrasts with INO caused by brainstem infarction which classically presents in older patients, with an average age of 62 to 66.^4^,^5^ These patients will usually have cardiovascular risk factors; hypertension, hyperlipidemia, diabetes, tobacco use. Studies by both Keane and Kim found that INO caused by cerebrovascular etiology will present unilaterally in up to 87 to 93% of cases.^3^,^4^ In patients with ischemic etiology, the INO may be isolated and be the only deficit; however, greater than 50% will have additional neurological deficits ranging from motor deficits, sensory deficits, dysarthria, facial paralysis, or gait ataxia.^4^

There are syndromes that have been described in association with an INO of ischemic etiology, most notably, “one-and-a-half syndrome” and “wall-eyed bilateral internuclear ophthalmoplegia (WEBINO),” which have more unique presentations.^13^,^14^ One-and-a-half syndrome describes an extensive pontine lesion that involves the MLF as well as either the cranial nerve VI nucleus, or the paramedian pontine reticular formation (PPRF) (Figure 1).^13^ The resultant deficient is a complete conjugate gaze palsy on the affected side with a concurrent INO.^13^ Only the contralateral eye can perform abduction; there is paresis of all other horizontal gaze functions. Wall-eyed bilateral internuclear ophthalmoplegia (WEBINO), given this name due to the resting bilateral exotropia that patients present with, occurs due to a lesion within the upper midbrain, which in addition to affecting the MLF pathway, also affects the convergence pathway.^14^ Therefore, in WEBINO syndrome, on exam patients will have resting exotropia, bilateral INO, and a loss of convergence, of which the latter is not typical for standard INO.^14^,^15^ Additionally, patients may have vertical nystagmus and or vertical gaze deficits.^14^

As noted in Table 1, after multiple sclerosis and cerebrovascular disease, the majority of other causes are anatomic owing to the course of the MLF, tracking just ventral to the fourth ventricle and cerebral aqueduct putting it at risk for compression from various etiologies. Additionally, and quite extraordinarily, there have been case reports in even mild head trauma causing INO.^15^,^16^ There are some important causes that are not anatomic, which become important to consider when imaging is negative for pathology. Wernicke’s encephalopathy, secondary to thiamine (vitamin B1) deficiency, presents as the triad of encephalopathy, oculomotor dysfunction, and gait ataxia. The oculomotor dysfunction is typically nystagmus, lateral rectus palsy, and conjugate gaze palsies which can present as an INO, although rare.^17^ Lastly, there are case reports of medications such as tricyclic antidepressants and phenothiazines antipsychotics inducing INO, which are thought to be in part related to anticholinergic side effects but the true mechanism is not well understood.^18^,^19^ There are also several other medications that have been implemented in causing drug-induced INO such as lithium and propranolol, but the latter was thought to be related to drug-drug interactions.^20^,^21^ Finally, there was a case report of INO being reversed with naloxone in the setting of narcotic use mixed with benzodiazepines, although the etiology in this case report was called into question.^22^,^23^

Clinicians should be aware of pseudo-internuclear ophthalmoplegias. These include partial CN III palsy, progressive supranuclear palsy, myasthenia gravis, and Guillain Barre syndrome (GBS).^3^,^24^–^29^ Patients with a partial CN III palsy will present with ptosis, mydriasis, weakness in elevation of the eyelid, and compared to a true INO patient, will have impaired convergence and a lack of nystagmus.^24^,^25^ Patients with progressive supranuclear palsy (PSP) will have Parkinsonian features, cognitive abnormalities, and the hallmark of impaired vertical gaze.^27^ This disease is progressive, as the name implies, and may take as long as ten years to fully develop. Hallmarks in terms of the oculomotor findings of PSP are slowing and limited vertical gaze, with concomitant limitations in lateral gaze which is why it can be mistaken for an INO.^24^,^27^ When ocular manifestations occur in myasthenia gravis and GBS, they usually occur in patients that have established diagnoses. Myasthenia gravis will present with bilateral ptosis and upper eyelid fatigue, and the extraocular muscles are often affected, resulting in binocular diplopia. Classically symptoms are worse later in the day or after use of these muscles.^28^,^30^ Guillain Barre syndrome classically presents with symmetric ascending paralysis with areflexia. Recall, however, the Miller Fischer Variant of GBS is classically a triad of ataxia, areflexia, and ophthalmoplegia.^29^ As always, it is important to consider the patient’s entire presentation, preceding symptoms, to obtain a detailed history, and do a detailed exam to get the most accurate diagnosis.

The diagnosis of an INO is made clinically with the impaired horizontal eye movements and abduction nystagmus of the contralateral eye.^24^ Work up should always include an MRI brain with and without contrast.^31^ If the MRI is normal, the clinician should focus on the nonstructural causes of INO as well as the various causes of pseudo-INO. However, there are reports of MRI not detecting a lesion that was still determined to be ischemic in etiology.^5^

Prognosis is variable and depends on the underlying etiology. In general, the deficits will resolve within months; however, they can be long standing.^4^–^6^ Studies are variable in terms of recovery in cerebrovascular etiology. Bolaños et al. reviewed 65 patients of various etiologies and found that 33 of the patients had persistent symptoms at greater than one year.^6^ Further analysis of this group revealed that 63 percent of the patients whose INO was secondary to cerebrovascular etiology still had some degree of persistent symptoms at three-year follow up.^6^ In contrast, both Kim and Eggenberger et al. demonstrated better prognosis with cerebrovascular etiology, with recovery of symptoms as quickly as within three months in 79 to 87 percent of patients.^4^,^5^ In Eggenberger’s et al. study, MRI demonstrated causative infarction in only 52 percent of cases; however, the other cases were still thought to be ischemic in etiology.^5^ The presence of an MRI identifiable ischemic lesion did not significantly correlate with resolution of symptoms; however, patients with other neurological deficits had worse prognosis in terms of time to resolution of INO.^5^ This was also observed by Kim, who noted that patients with associated neurologic deficits were more likely to have longer lasting INO; however, the INO eventually resolved in all patients.^4^

Unfortunately, unless there is a relatively reversible cause such as multiple sclerosis, there is no promising treatment for INO. In cases of cerebrovascular etiology, it is important to modify risk factors to reduce future stroke risk. Patients with severe diplopia can be treated with patching of one eye. Prism lenses are generally not helpful, but options can be explored in consultation with ophthalmology.

The strengths of our patient approach were the basis of good history and physical exam to secure our diagnosis. The MRI then confirmed the etiology, which was surprising in a seemingly healthy 40-year-old. There were no perceived limitations to our patient approach or work up; however, our physical exam could have been improved by ensuring convergence was intact. This case and our discussion highlights how the presenting symptoms of an INO may be subtle such as the complaint of vertigo, as in our patient. Furthermore, it demonstrates the importance of taking a detailed history and physical exam, which will reveal the deficit of an INO. Our case report was strengthened by review of the current literature on INO. One limitation of our case report was the older case reports of medications/drugs causing INO having a low “*n”* and may not be generalizable. We hope to have added to the literature by demonstrating that patients do not always follow the general rules. When an INO is discovered, an MRI with and without contrast should always be obtained to help discern the etiology.

## Supplementary Information















## Figures and Tables

**Table 1:** Differential diagnosis of INO^3^,^4^,^15^–^22^

**Multiple Sclerosis**	Often bilateral, younger patient, usually <45 years old
**Brainstem Infarction**	Often unilateral, older patient, usually >60 years old with cardiovascular risk factors
**Other Causes:**	
Anatomical	Brainstem tumor, fourth ventricle tumor, Arnold Chiari malformation, arteriovenous malformation, hydrocephalus
Trauma	Subdural hematoma, head trauma
Infection/Inflammatory	Meningoencephalitis, syphilis, vasculitis
Encephalopathy	Wernicke’s encephalopathy, hepatic encephalopathy
Medications/Drugs	Tricyclic antidepressants, phenothiazines, lithium, narcotics, barbiturates, propranolol

## References

[1] Herr RD, Zun L, Mathews JJ (1989). A directed approach to the dizzy patient. Ann Emerg Med.

[2] Frohman EM, Zhang H, Kramer PD (2001). MRI characteristics of the MLF in MS patients with chronic internuclear ophthalmoparesis. Neurology.

[3] Keane JR (2005). Internuclear ophthalmoplegia: unusual causes in 114 of 410 patients. Arch Neurol.

[4] Kim JS (2004). Internuclear ophthalmoplegia as an isolated or predominant symptom of brainstem infarction. Neurology.

[5] Eggenberger E, Golnik K, Lee A (2002). Prognosis of ischemic internuclear ophthalmoplegia. Ophthalmology.

[6] Bolaños I, Lozano D, Cantú C (2004). Internuclear ophthalmoplegia: causes and long-term follow-up in 65 patients. Acta Neurol Scand.

[7] Frohman TC, Galetta S, Fox R (2008). Pearls & Oy-sters: The medial longitudinal fasciculus in ocular motor physiology. Neurology.

[8] Blumenfeld H (2018). Neuroanatomy through Clinical Cases.

[9] Mills DA, Frohman TC, Davis SL (2008). Break in binocular fusion during head turning in MS patients with INO. Neurology.

[10] Zee DS, Hain TC, Carl JR (1987). Abduction nystagmus in internuclear ophthalmoplegia. Ann Neurol.

[11] Zee DS (1992). Internuclear ophthalmoplegia: pathophysiology and diagnosis. Baillieres Clin Neurol.

[12] Bronstein AM, Rudge P, Gresty MA, Du Boulay G, Morris J (1990). Abnormalities of horizontal gaze. Clinical, oculographic and magnetic resonance imaging findings. II. Gaze palsy and internuclear ophthalmoplegia. J Neurol Neurosurg Psychiatry.

[13] Wall M, Wray SH (1983). The one-and-a-half syndrome--a unilateral disorder of the pontine tegmentum: a study of 20 cases and review of the literature. Neurology.

[14] Wu YT, Cafiero-Chin M, Marques C (2015). Wall-eyed bilateral internuclear ophthalmoplegia: review of pathogenesis, diagnosis, prognosis and management. Clin Exp Optom.

[15] Jung DS, Park KP (2004). Posttraumatic bilateral internuclear ophthalmoplegia with exotropia. Arch Neurol.

[16] Constantoyannis C, Tzortzidis F, Papadakis N (1998). Internuclear ophthalmoplegia following minor head injury: a case report. Br J Neurosurg.

[17] Chandrakumar A, Bhardwaj A, ‘t Jong GW (2018). Review of thiamine deficiency disorders: Wernicke encephalopathy and Korsakoff psychosis. J Basic Clin Physiol Pharmacol.

[18] White A (1988). Overdose of tricyclic antidepressants associated with absent brain-stem reflexes. CMAJ.

[19] Cook FF, Davis RG, Russo LS (1981). Internuclear ophthalmoplegia caused by phenothiazine intoxication. Arch Neurol.

[20] Deleu D, Ebinger G (1989). Lithium-induced internuclear ophthalmoplegia. Clin Neuropharmacol.

[21] Cunningham GM (1983). Drug-induced internuclear ophthalmoplegia. Can Med Assoc J.

[22] Rizzo M, Corbett J (1983). Bilateral internuclear ophthalmoplegia reversed by naloxone. Arch Neurol.

[23] Gillman MA, Sandyk R (1984). Bilateral internuclear ophthalmoplegia reversed by naloxone. Arch Neurol.

[24] Ranalli PJ, Sharpe JA (1988). Vertical vestibulo-ocular reflex, smooth pursuit and eye-head tracking dysfunction in internuclear ophthalmoplegia. Brain.

[25] Bhatti MT, Eisenschenk S, Roper SN, Guy JR (2006). Superior divisional third cranial nerve paresis: clinical and anatomical observations of 2 unique cases. Arch Neurol.

[26] Flint AC, Williams O (2005). Bilateral internuclear ophthalmoplegia in progressive supranuclear palsy with an overriding oculocephalic maneuver. Mov Disord.

[27] Respondek G, Kurz C, Arzberger T (2017). Which ante mortem clinical features predict progressive supranuclear palsy pathology?. Mov Disord.

[28] Grob D, Brunner N, Namba T, Pagala M (2008). Lifetime course of myasthenia gravis. Muscle Nerve.

[29] Fisher M (1956). An unusual variant of acute idiopathic polyneuritis (syndrome of ophthalmoplegia, ataxia and areflexia). N Engl J Med.

[30] Oosterhuis HJ (1989). The natural course of myasthenia gravis: a long term follow up study. J Neurol Neurosurg Psychiatry.

[31] Atlas SW, Grossman RI, Savino PJ (1987). Internuclear ophthalmoplegia: MR-anatomic correlation. AJNR Am J Neuroradiol.

