# Supplementary figures and images for: The Clue is in the Eyes. A Case Report of Internuclear Ophthalmoplegia
Source: J Educ Teach Emerg Med. 2024 Jan 31;9(1):V1–8. doi: 10.21980/J8DP9M (PMC10854879; doi:10.21980/J8DP9M)

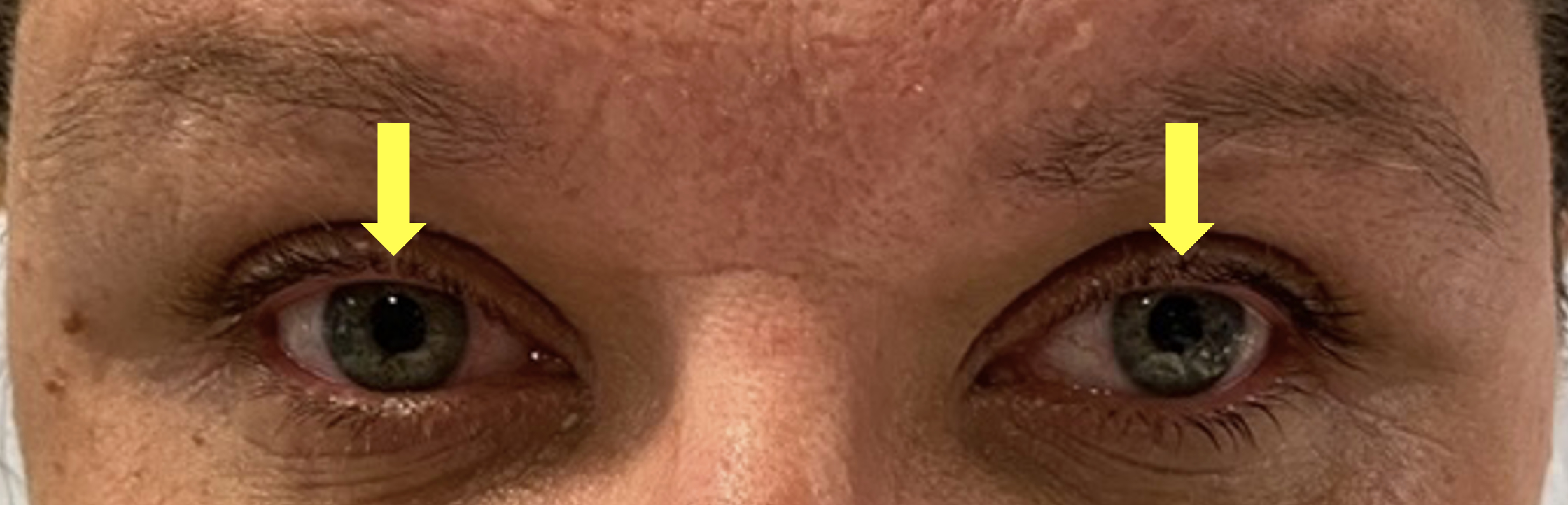

Supplement: Supplementary file 1 [file jetem-9-1-V1-supp1.jpeg]

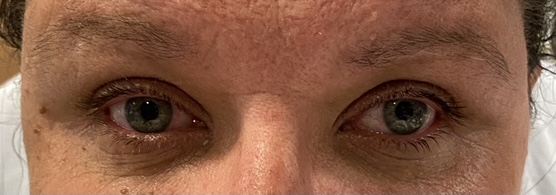

Supplement: Supplementary file 2 [file jetem-9-1-V1-supp2.jpeg]

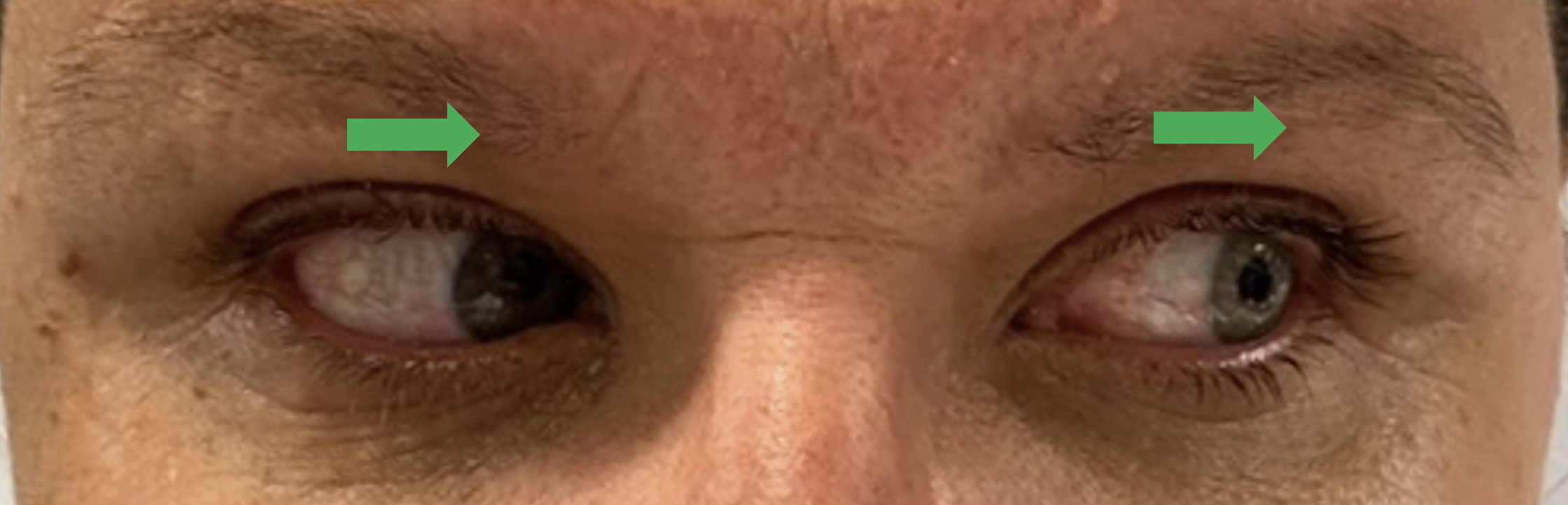

Supplement: Supplementary file 3 [file jetem-9-1-V1-supp3.jpg]

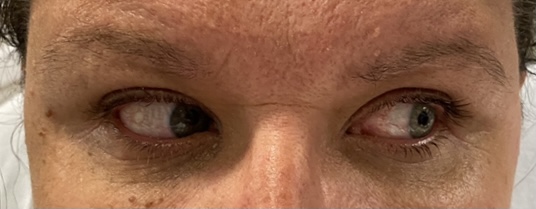

Supplement: Supplementary file 4 [file jetem-9-1-V1-supp4.jpeg]

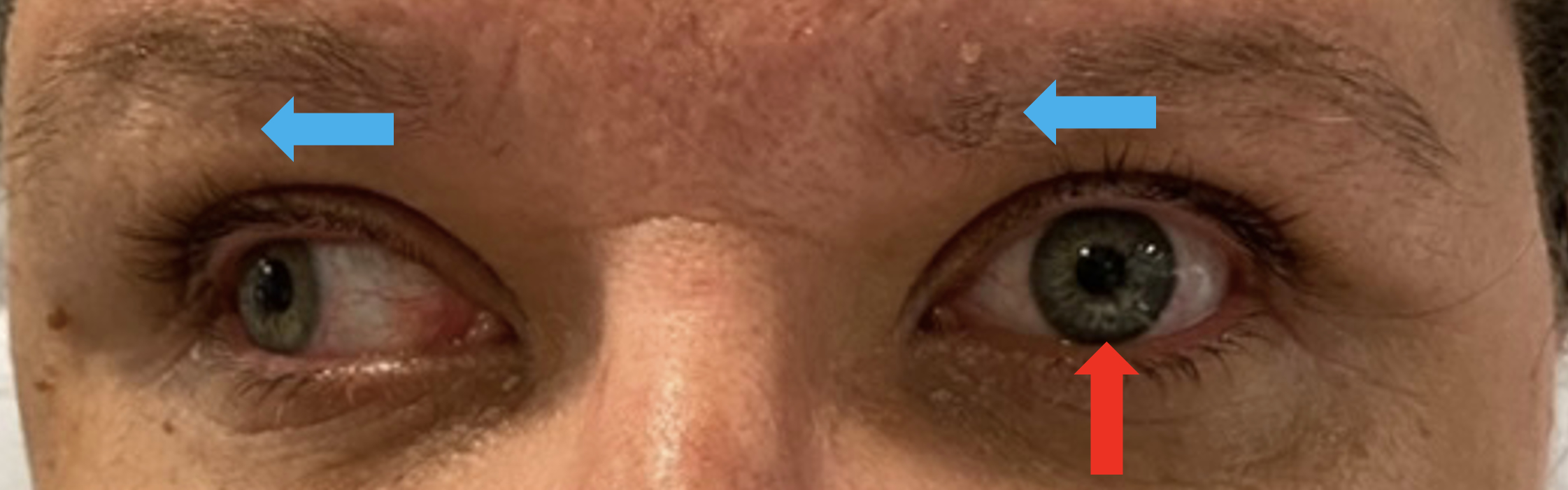

Supplement: Supplementary file 5 [file jetem-9-1-V1-supp5.jpg]

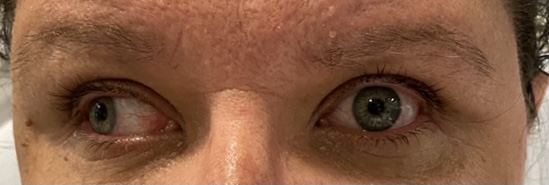

Supplement: Supplementary file 6 [file jetem-9-1-V1-supp6.jpeg]

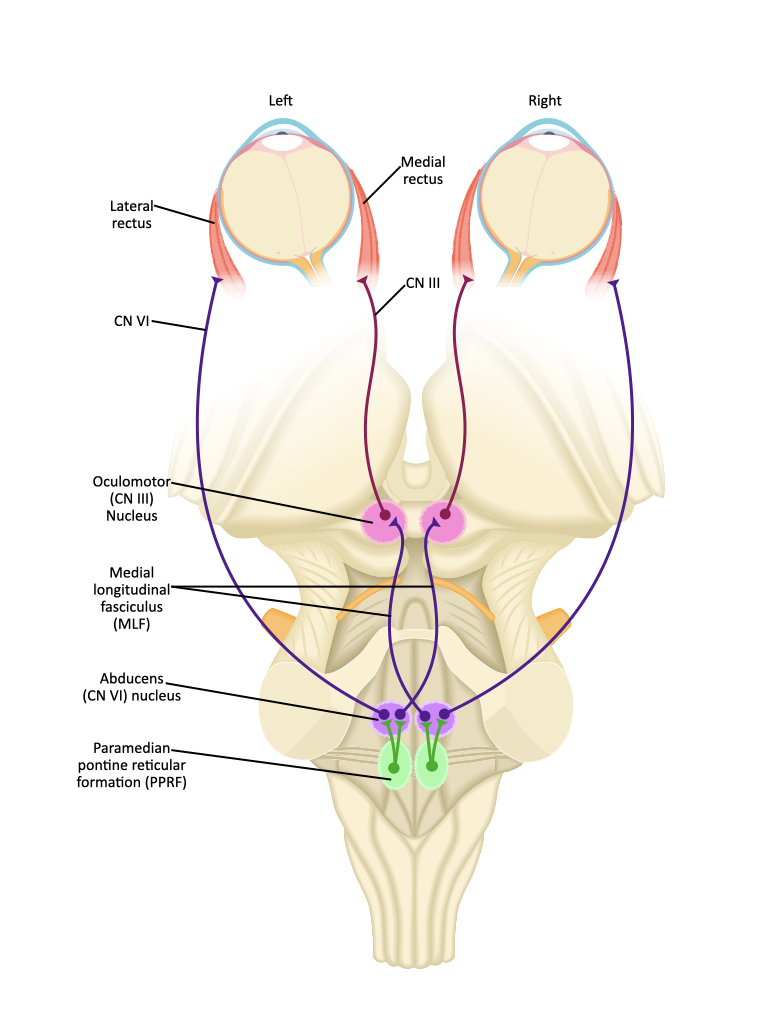

Supplement: Supplementary file 7 [file jetem-9-1-V1-supp7.png]
